# Six-Month Quality of Life and Health Outcomes After Bariatric Surgery: A Prospective Cohort Study from Latvia

**DOI:** 10.3390/medicina61122238

**Published:** 2025-12-18

**Authors:** Guna Bīlande, Marina Arisova, Maksims Mukāns, Igors Troickis

**Affiliations:** 1 Aiwa Clinic, LV-1019, Riga, Latvia; 2 Faculty of Medicine and Life Sciences, University of Latvia, LV-1004 Riga, Latvia; 3 Faculty of Health Care, Turība University, LV-1058 Riga, Latvia; 4 Statistics Unit, Riga Stradiņš University, LV-1048 Riga, Latvia

**Keywords:** bariatric surgery, obesity, quality of life (QoL), Bariatric Quality of Life (BQL), weight loss

## Abstract

*Background and Objectives*: Obesity is a major public health concern associated with reduced quality of life (QoL) and multiple comorbidities. Bariatric surgery is an effective treatment for severe obesity; however, postoperative QoL outcomes in Latvia remain insufficiently studied. This prospective study evaluated short-term changes in QoL, weight loss, and selected health parameters six months after bariatric surgery. *Materials and Methods*: Data were collected from 17 adults who underwent bariatric surgery at a single centre in Riga, Latvia. QoL was assessed preoperatively and six months postoperatively using the validated Bariatric Quality of Life (BQL) questionnaire. Anthropometric measurements, self-reported comorbidities, and medication use were obtained at both time points. Statistical analysis was performed using non-parametric methods (*p* < 0.05). *Results*: Participants had a median age of 54 years, and 76% were female. Six months after surgery, significant reductions were observed in BMI (39.7 to 31.6 kg/m^2^; *p* < 0.0001), total weight (−23.3%), and excess weight (−60.7%). The total BQL score increased from 44.5 to 52.0 points (*p* = 0.004), indicating improved QoL. Self-reported hypertension and sleep apnoea decreased, and all individuals with preoperative reflux symptoms reported resolution. Waist circumference declined but often remained above metabolic risk thresholds. Hair loss was the most frequently reported postoperative adverse effect. *Conclusions*: Bariatric surgery was associated with short-term improvements in QoL, weight loss, and several obesity-related symptoms. Hair loss was common but expected. Given the small sample size and single-centre design, findings should be interpreted as exploratory. Larger studies with longer follow-up are needed to better characterize long-term outcomes and support the development of bariatric care in Latvia.

## 1. Introduction

Obesity has become a significant public health challenge worldwide, with profound health and economic consequences. The economic burden of obesity is considerable across diverse countries, irrespective of their economic status or geographical location, and is expected to escalate over time if current trends persist [1]. According to the World Health Organization, it is projected that by 2030, approximately 60% of the global population will be affected by overweight or obesity, with 1.1 billion individuals classified as obese [2].

With global prevalence of obesity increasing year on year, and corresponding increases in the burden of related disease, it is clear that obesity is a problem that requires urgent attention [3].

Obesity is a chronic noncommunicable disease, defined by a body mass index (BMI) exceeding 30 kg/m^2^. Its impact extends beyond its association with increased risks of mortality and morbidity from other noncommunicable diseases, and also affects quality of life (QoL) [4].

It is known that bariatric surgery is an effective and sustainable method of weight loss, resulting in reduced risk and severity of comorbidities such as type 2 diabetes, dyslipidemia, non-alcoholic fatty liver disease, gastroesophageal reflux disease (GERD), hypertension, polycystic ovarian syndrome, sleep apnoea and arthrosis [5,6,7,8]. The primary benefits of weight reduction following bariatric surgery include not only improvements in comorbidities, but also enhanced quality of life, and a reduction in all-cause mortality [9]. Studies have shown improved functional capacity and increased activity levels in patients after bariatric surgery [10].

QoL has been recognized as an important marker of health for the general population and those with chronic or life-threatening conditions. Patients seek surgical care most often because of impaired QoL, and thus improvement in QoL serves as a key indicator for assessing the effectiveness of the treatment [11].

In 2022, 36.1% of the population in Latvia was classified as overweight, while 23.3% were affected by obesity. This trend has shown a consistent increase over last years [12].

The number of bariatric surgeries performed in Latvia has been increasing; however, this growth remains disproportionate to the prevalence of obesity in the population. Bariatric surgery continues to be underutilized in Latvia, with limited research available on the effectiveness of this treatment and its impact on the QoL among Latvian patients. For example in 2015, a total of 276 bariatric surgeries were performed, rising to 593 by 2019. Despite some public resistance, the expertise and professionalism of Latvian surgeons have been highly regarded by international patients. Notably, 78% (*n* = 460) of the 593 bariatric surgeries performed in 2019 were for foreign patients. Severe obesity and its related comorbidities, such as type 2 diabetes, hypertension, dyslipidemia, and sleep apnoea, are prevalent in Latvia; however, a relatively small number of patients with these conditions opt for bariatric surgery. This is largely due to the high out-of-pocket expenses associated with the procedure, which presents significant accessibility concerns [13].

The aim of this study was to assess the dynamics of QoL and health status among bariatric surgery patients in Latvia over a six-month period following surgery. This study is the first study of this kind conducted in a Latvian cohort.

## 2. Materials and Methods

This was a quantitative, descriptive, and longitudinal study involving repeated measurements within the same patient cohort over time. The study was conducted at AIWA Clinic, a multidisciplinary surgical private clinic in Riga, Latvia, between March 2021 and December 2021, as part of the FLPP project “Impact of Weight Reduction on B-Cell Metabolism and Function”.

QoL was performed using the Bariatric Quality of Life (BQL) Index, a validated questionnaire specifically designed for individuals undergoing bariatric surgery. The BQL assesses both bariatric-specific and health-related quality of life (HRQoL) domains within a single instrument. It was originally developed in 2005 and updated in 2009 by Weiner et al. [14,15]. The questionnaire consists of 30 items; higher total scores indicate a better QoL. The BQL was translated into Latvian and subsequently validated. All questionnaires were provided in Latvian with verbal instructions. Completion time was approximately 20–25 min per participant.

Participants completed the QoL questionnaire at two time points: prior to bariatric surgery and six months post-surgery. At baseline participants underwent anthropometric measurements, including body weight, waist circumference, hip circumference, and height. In addition, demographic information such as age, gender, education level, occupation, and marital status was collected. Preoperative evaluation was performed by a multidisciplinary team and included assessment of medical history, physical examination, and relevant laboratory tests. At the 6-month follow-up, participants were reassessed with repeated measurements of body weight, waist circumference, and hip circumference.

Data were analyzed using IBM SPSS Statistics version 27. All analyses were performed using a per-protocol approach, including only participants with complete baseline and six-month follow-up data. Continuous variables were assessed for normality using the Shapiro–Wilk test. Because most variables violated normality assumptions and the sample size was small, non-parametric statistical methods were applied.

Descriptive statistics are presented as medians with interquartile ranges (Me, IQR) for continuous variables and as frequencies and percentages for categorical variables. Pre- and postoperative differences in anthropometric measures, QoL scores, and health-related parameters were examined using the Wilcoxon signed-rank test.

Associations between continuous variables (e.g., BMI, %EWL, QoL, HRQoL) were evaluated using Spearman’s rank correlation coefficient (ρ). Differences between surgical subgroups Roux-en-Y gastric bypass (RYGB) vs. vertical sleeve gastrectomy (VSG) were explored using the Mann–Whitney U test, although due to the small number of VSG cases, subgroup analyses were interpreted cautiously.

Categorical variables, including presence/absence of comorbidities (hypertension, sleep apnoea, GERD, joint pain, etc.), were compared before and after surgery using the McNemar test or binomial test, depending on expected frequencies.

No imputation was used for missing data; only complete cases were included. Statistical significance was set at *p* < 0.05 for all analyses. Graphs and tables were produced using Microsoft Excel 2019.

Due to the small sample size, the study was underpowered for detecting small effect sizes, and findings should be interpreted as exploratory.

## 3. Results

The study sample consisted of 21 adults (aged 18 years or older) residing in the Republic of Latvia, all diagnosed with either morbid obesity (body mass index (BMI) ≥ 40 kg/m^2^) or severe obesity (BMI ≥ 35 kg/m^2^) accompanied by at least one obesity-related comorbidity. Throughout the study, four participants did not complete the six-month follow-up: two were lost to follow-up due to relocation, one withdrew consent, and one was unable to attend planned consultations. No postoperative complications were reported among participants who discontinued follow-up.

**Figure 1:**
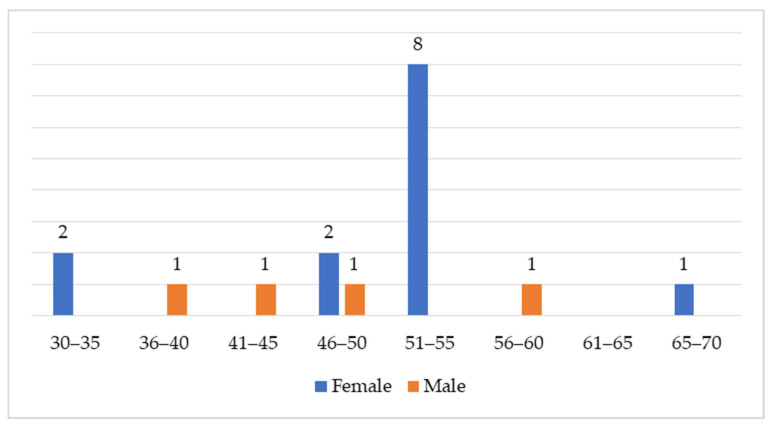
The distribution of participants by gender and age.

Of the 17 participants included in the final analysis, four were male and 13 were female. The median age was 54 years (IQR: 46–55), with the youngest participant being 34 years and the oldest 66 years (Figure 1).

The majority of participants had higher education (12 individuals), four had vocational education, and one had secondary education. Regarding marital status, 11 participants were married, one was in a relationship, two were divorced, and three lived alone.

**Figure 2:**
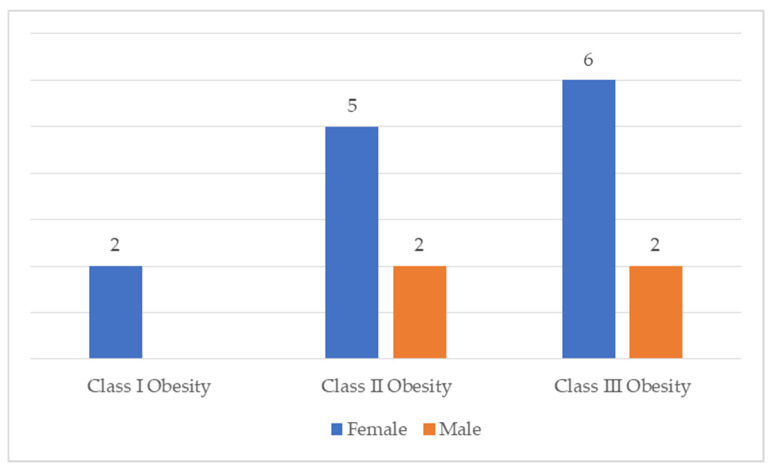
The distribution of participants across weight categories and genders.

At the beginning of the study, all participants had obesity ranging from class I to class III (Figure 2). The median body mass index (BMI) at baseline was 39.68 kg/m^2^ (IQR: 36.29–44.62).

The median preoperative weight was 115 kg (IQR: 97.00–132.50), with the minimum weight being 87 kg and the maximum weight 179 kg. The median waist circumference was 125 cm (IQR: 107.75–134.25), with the minimum waist circumference measured at 91 cm and the maximum at 143 cm.

The most prevalent preoperative health conditions included: nine participants had hypertension, six experienced heartburn, three had acid reflux, eight reported joint pain, five had sleep apnoea, and four experienced urinary incontinence. Additionally, eight participants had gallstones, one had type 2 diabetes, and two reported preoperative hair loss. Ten participants reported regular use of medications, including eight for blood pressure management, two for pain relief, two for antidepressants, and one antidiabetic medication. Regarding weight loss prior to surgery, none of the participants reported weight reduction in the three months preceding the procedure.

Among the 17 participants, 15 underwent RYGB and 2 VSG. No statistically significant differences were observed between the two surgical groups; therefore, their results were combined for all analyses in this exploratory study.

Six months after surgery, reductions were observed in all participants in weight, BMI, and waist circumference (see Table 1).

BMI decreased from a median of 39.68 kg/m^2^ to 31.62 kg/m^2^ (*p* < 0.0001). Weight decreased from 115.00 kg to 88.00 kg (*p* < 0.0001). Waist circumference decreased from 125.50 cm to 103.00 cm (*p* < 0.001).

The participant with the least weight reduction (−7.0 kg) transitioned from class II to class I obesity six months after bariatric surgery. In contrast, the participant with the greatest weight reduction (−72.3 kg) moved from class III obesity to the overweight category. The maximum total weight loss (%TWL) was 40.39% of initial body weight, whereas the minimum %TWL was 7.07%. The maximum excess weight loss (%EWL) reached 107.75%, and the minimum %EWL was 22.78%.

At six months postoperatively, 14 participants (82% of the cohort) achieved an excess weight loss of ≥50%, a threshold commonly used to define successful bariatric outcomes. However, this proportion reflects only the current exploratory cohort and should not be generalized.

No statistically significant correlations were observed between baseline BMI and QoL measures, nor between the magnitude of weight loss and postoperative improvements in QoL. Similarly none of the weight loss indicators examined showed a significant relationship with HRQoL change; this included %EWL (r = 0.03, *p* = 0.909), %TWL (r = –0.015, *p* = 0.954), and absolute weight loss (r = –0.02, *p* = 0.939). A moderate positive correlation was observed between overall QoL change and self-reported engagement in physical activity (r = 0.469), although this trend did not reach statistical significance (*p* = 0.058).

With respect to gastroesophageal reflux symptoms, all six participants who reported preoperative GERD underwent RYGB, and none reported persistent GERD symptoms at the six-month follow-up. No GERD cases were observed among participants who underwent sleeve gastrectomy.

**Figure 3:**
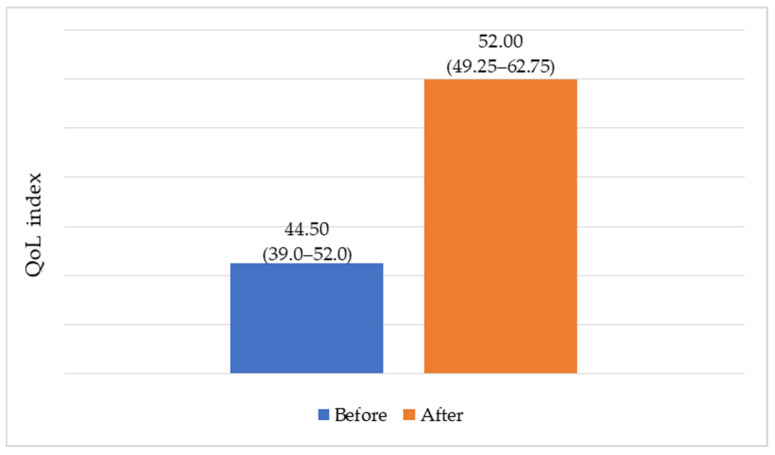
The changes in QoL index (Me (IQR)).

Overall, six months after bariatric surgery, a positive trend was observed in the overall QoL index, which increased by 17% over this relatively short period (*p* = 0.004) (Figure 3).

**Figure 4:**
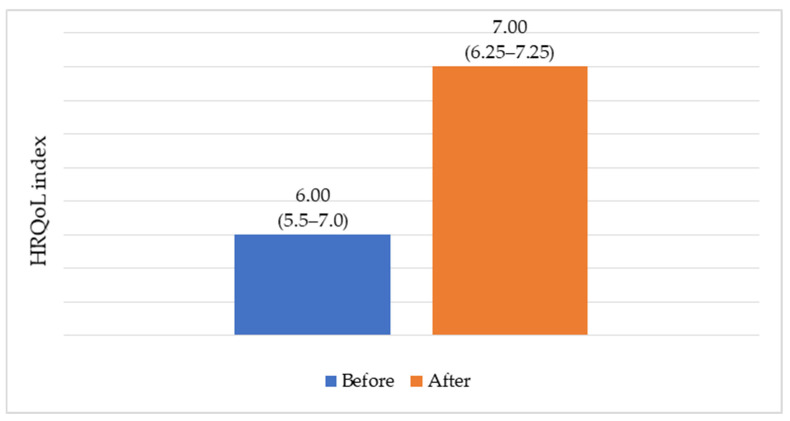
The changes in HRQoL index (Me (IQR)).

Six months post-surgery, the HRQoL index increased by 17% (*p* = 0.044), reaching a median score of 7 (IQR: 6.25–7.25). This median score was approximately 13%, below the maximum achievable HRQoL value of 8 points. Additionally, the minimum absolute HRQoL score improved by one point, increasing from 4.5 to 5.5. The highest observed values approached the upper range of the HRQoL scale. The changes in HRQoL before and after surgery are illustrated in Figure 4.

**Figure 5:**
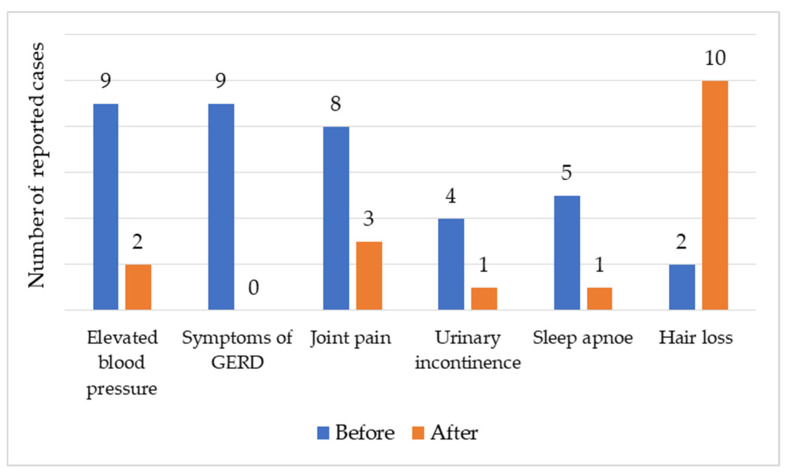
The dynamics of the reported health conditions before and after surgery.

Six months post-surgery, significant changes were observed in several health parameters. Blood pressure showed a statistically significant reduction (*p* = 0.016), while symptoms of GERD, joint pain (*p* = 0.063), and sleep apnoea (*p* = 0.125) exhibited changes that did not reach statistical significance. Conversely, the frequency of self-reported hair loss increased (*p* = 0.008). The dynamics of the reported health conditions before and after surgery are presented in Figure 5.

The results of the health status assessment were utilized to calculate the HRQoL index, which constitutes one of the subscales in the bariatric surgery patient quality of life evaluation questionnaire.

**Figure 6:**
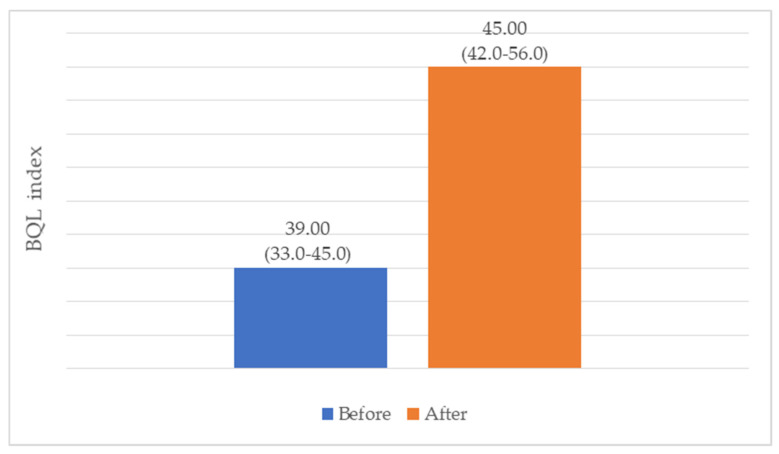
Changes in BQL index (Me (IQR)).

The second section of the questionnaire aims to assess the non-health-related QoL index. A comparison of the results before and six months after the procedure reveals a 15% increase in the BQL index, with a median value of 45 (IQR: 42–56) (*p* = 0.006) (see Figure 6). These values remained below the upper range of the instrument, indicating higher scores within the cohort but not approaching ceiling levels.

Analysis of the responses to the 14 questions revealed the largest quantitative changes and statistically significant differences in the following statements:

1. “I like my weight” (*p* = 0.003);
2. “I can accept my weight” (*p* = 0.006);
3. “How is your actual quality of life?” (*p* = 0.002);
4. “All in all, I feel satisfied in my life” (*p* = 0.024);
5. “I feel restricted because my weight privately” (*p* = 0.012).

**Figure 7:**
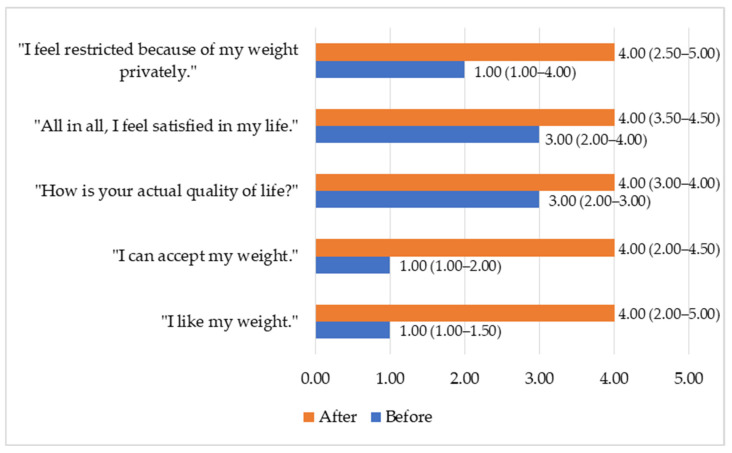
Items of BQL subscale of QoL (Me (IQR).

Responses to these questions were evaluated using a five-point Likert scale, where the highest score indicated “Strongly agree” and the lowest score indicated “Strongly disagree” (Figure 7). Higher scores at follow-up reflect higher item ratings within this cohort and should not be interpreted as generalizable effects.

Positive changes were observed in participants’ attitudes toward physical activity. Prior to bariatric surgery, 35% of patients selected “Mostly agree” or “Strongly agree” to the statement “I exercise regularly.” Six months post-surgery, this percentage increased to 53%. Minimal changes were observed in other questionnaire items.

## 4. Discussion

This exploratory study assessed changes in quality of life, weight loss, and selected health parameters six months after bariatric surgery in a Latvian patient cohort. Attrition was low (19%), although it remains possible that participants who discontinued follow-up differed in postoperative progress or quality-of-life trajectories.

Participants demonstrated statistically significant increases in both overall QoL and HRQoL scores at six months compared with baseline. Weight reduction was substantial, with a median %EWL of 60.65% and %TWL of 23.31%. Several obesity-related conditions, including hypertension and sleep apnoea, were reported less frequently postoperatively, whereas hair loss was reported more often.

The QoL improvements observed in this cohort are consistent with findings from Mocian and Coroș, who reported significant increases in BQL scores one year after bariatric surgery using the same instrument [16]. Robertson et al. similarly identified statistically significant QoL improvements during the first six postoperative months, which were maintained at 12 months [17].

Similar conclusions have been reported in a recent narrative review by Alharbi et al., who found that bariatric surgery consistently improves patient satisfaction and overall quality of life across physical, psychological, and social domains, despite variations in study design and follow-up duration [18].

In this study, no significant associations were found between the magnitude of weight loss and improvements in QoL or HRQoL. These findings suggest that early postoperative QoL gains may be influenced by factors other than absolute weight reduction, such as symptom relief, improved mobility, and psychological expectations, just the same patterns that have also been reported in previous research. A moderate correlation was observed between QoL improvement and engagement in physical activity (r = 0.469, *p* = 0.058). This may indicate that increased physical activity contributes to participants’ perceived well-being, although confirmation in larger samples is required.

Improvements in satisfaction with weight and weight acceptance observed in the present study are also in line with findings by Mocian and Coroș, who reported the lowest preoperative ratings in these domains and marked postoperative improvements [16]. These results are coherent with broader evidence that individuals with obesity frequently experience lower body image compared with the wider population, and that satisfaction with body weight tends to improve after substantial weight loss.

Nickel et al. also demonstrated that QoL, body image, and general self-efficacy significantly improved within six months after bariatric surgery and remained stable at 24 months, independent of demographic or surgical factors [19]. The absence of a correlation between BMI and QoL in the present study aligns with the hypothesis by Meneguzzo et al., who suggested that self-image evaluation among bariatric patients may be partly independent of actual BMI and more influenced by psychological and perceptual factors [20].

Weight loss outcomes at six months were similar to those reported by Mahdy et al. (%EWL 50.8 ± 20.6%) and D’Eusebio et al. (%EWL 54.8 ± 33.0%) [21,22]. The median %TWL of 23.31% in this cohort is also comparable to the 24 ± 7.5% total weight loss reported by Mahdy et al. [21]. Given that even modest reductions of 7–11% in total body weight have been associated with improvements in the severity of hypertension and sleep apnoea, and losses ≥10% may alleviate GERD symptoms [23], the weight loss magnitude observed in this study is clinically meaningful. All participants with preoperative GERD who underwent RYGB reported complete symptom resolution at six months, which is consistent with evidence indicating that gastric bypass is particularly effective in reducing reflux symptoms.

Five participants reported sleep apnoea preoperatively, whereas only one participant reported persisting symptoms six months postoperatively. Similar observations have been described in the literature, where weight reduction after bariatric surgery contributes to improvements in sleep apnoea severity. Hypertension also decreased substantially in this cohort, with a 70% reduction in self-reported hypertension and a 38% reduction in antihypertensive medication use. Comparable reductions have been reported by Mocian and Coroș (70% at one year) and by Hussain and El-Hasani, who reported reductions of 61%, 58%, and 58% in the first three postoperative years [16,24].

Reflux symptoms also improved. Six patients reported preoperative heartburn or acid regurgitation, and none reported these symptoms six months after surgery. This finding is consistent with the literature indicating that RYGB is highly effective for resolving GERD symptoms. A systematic review by Ashraf, Osland, and Memon documented significant reductions in heartburn and regurgitation following gastric bypass across multiple studies [25]. Given that all six symptomatic patients underwent RYGB, this likely contributed to symptom resolution. However, objective diagnostic testing was not performed, and therefore results should be interpreted cautiously.

Evidence regarding GERD after bariatric surgery highlights the importance of careful patient selection, as surgery may both alleviate and induce reflux depending on the procedure type [25,26,27]. In contemporary literature, RYGB remains the most reliably effective procedure for GERD resolution, while VSG is associated with higher rates of de novo or persistent reflux [26,27]. This context underscores the relevance of evaluating reflux symptoms preoperatively—a practice that was part of the standard preoperative assessment in this study.

Reductions in joint pain were also observed, although three participants continued to report persistent symptoms. Similar findings were noted by Mocian and Coroș, where only nine of 28 patients continued to experience joint pain one year after surgery [16]. Kerver et al. reported that general body aches, particularly those associated with osteoarthritis, tend to decrease following bariatric surgery, but outcomes vary based on weight loss magnitude and the presence of irreversible joint damage [28]. The limited improvement in some participants in this study may reflect insufficient time for musculoskeletal recovery, inadequate weight loss for symptom relief, long-standing joint pathology, or increased physical activity after surgery, as more participants reported regular exercise at six months. Heuts et al. highlighted similar associations between increased activity and postoperative joint discomfort [29].

Hair loss was reported more frequently at six months than at baseline. This finding is consistent with prior research demonstrating that telogen effluvium is a common postoperative occurrence linked to rapid weight loss, micronutrient deficiencies, reduced protein intake, and anesthesia-related stress [30,31]. Symptoms typically begin within 2–3 months after surgery and tend to resolve within six months. Although two patients reported preoperative hair loss, possibly due to pre-existing micronutrient deficiencies, dietary intake data were not collected, limiting further analysis.

Published evidence on bariatric surgery outcomes in Latvia remains extremely limited [13,32]. This study therefore provides one of the first prospective clinical observations of postoperative QoL and short-term health changes in a Latvian context. The cohort’s demographic profile—predominantly middle-aged women with higher education—may reflect the population able to access self-funded bariatric procedures, as public funding for bariatric surgery in Latvia is limited. This context may influence patient characteristics, expectations, and postoperative experiences and highlights the need for broader national data.

The study has several limitations. The sample size was small and drawn from a single private clinic, limiting generalizability. The study was underpowered to detect smaller differences, and results should be interpreted cautiously. Comorbidities were partly self-reported, and no objective diagnostic testing (e.g., sleep studies or endoscopy) was used to confirm conditions. Follow-up duration was limited to six months, which may not capture the full postoperative trajectory. Additional sociodemographic data, such as income level or rural versus urban residence, were not collected.

Despite these limitations, the findings provide preliminary evidence that bariatric surgery in Latvia is associated with improvements in QoL and reductions in selected obesity-related symptoms. Larger, multicenter prospective studies with extended follow-up and broader patient characterization are needed to confirm these results. Establishing a national bariatric outcomes registry may support systematic postoperative monitoring and strengthen evidence based decision-making in Latvia.

## 5. Conclusions

In this exploratory cohort, bariatric surgery was associated with meaningful short-term improvements in weight, obesity-related symptoms, and quality of life. At six months, patients demonstrated significant increases in overall QoL and HRQoL scores and achieved clinically relevant weight reduction, with most reaching satisfactory excess weight loss. Improvements in hypertension, sleep apnoea, and reflux symptoms were also reported, consistent with international evidence on early metabolic benefits of bariatric procedures. Joint pain decreased in several participants, although persistent symptoms suggest that some effects may depend on pre-existing joint status or insufficient weight loss. Hair loss was a common but expected short-term postoperative effect, underscoring the need for structured nutritional follow-up.

Given the small sample size and single-centre design, these findings should be interpreted cautiously and cannot be generalized to the broader Latvian bariatric population.

Larger studies with extended follow-up are needed to evaluate long-term outcomes through comprehensive perioperative and postoperative care.

## Figures and Tables

**Table 1:** Weight and waist circumference changes amongst bariatric patients.

	Before the Surgery Me (IQR)	6 Months After the Surgery Me (IQR)	*p*
BMI (kg/m^2^)	39.68 (36.29–44.62)	31.62 (27.56–33.91)	<0.0001
Weight (kg)	115.00 (97.00–132.50)	88.00 (72.80–103.50)	<0.0001
Weight loss (kg)		26.50 (23.00–32.00)	
%TWL *		23.31 (19.84–27.25)	
%EWL **		60.65 (51.87–82.01)	
Waist (cm)	125.50 (107.75–134.25)	103.00 (93.50–114.00)	<0.001
Circumference lost on waist (cm)		23.00 (14.50–25.25)	

* %TWL—percentage of total weight loss; ** %EWL—percentage of excess weight loss.

## Data Availability

The datasets used and/or analyzed during the current study are available from the corresponding author on reasonable request.

## Abbreviations

The following abbreviations are used in this manuscript:

QoL	Quality of life
BQL	Bariatric quality of life
HRQoL	Health-related quality of life
GERD	Gastroesophageal reflux disease
RYGB	Roux-en-Y gastric bypass
VSG	vertical sleeve gastrectomy
%TWL	percentage of total weight loss
%EWL	percentage of excess weight loss
